# Skin and Induced Pluripotent Stem Cells as Biomarkers for Neurodegenerative Diseases

**DOI:** 10.3390/genes15121507

**Published:** 2024-11-25

**Authors:** Efstathios Rallis, Vasiliki-Sofia Grech, Kleomenis Lotsaris, Niki Tertipi, Eleni Sfyri, Vassiliki Kefala

**Affiliations:** 1 Department of Biomedical Sciences, School of Health and Care Sciences, University of West Attica, GR-12243 Athens, Greece; erallis@uniwa.gr (E.R.); ntertipi@uniwa.gr (N.T.); elsfiri@uniwa.gr (E.S.); valiakef@uniwa.gr (V.K.); 2 Psychiatrist in Department of Psychiatry, Athens General Hospital ‘Evaggelismos’, GR-10676 Athens, Greece; psych.kleolots@gmail.com

**Keywords:** neurodegenerative diseases, skin, fibroblasts, genes, proteins, iPSc

## Abstract

As the global population ages, the rising prevalence of neurodegenerative diseases, characterized by abnormal protein aggregates, presents significant challenges for early diagnosis and disease monitoring. Identifying accessible tissue biomarkers is crucial for advancing our ability to detect and track the progression of these diseases. Among the most promising biomarkers is the skin, which shares a common embryological origin with the brain and central nervous system (CNS). This biological connection positions the skin as a potential reflection of CNS pathology. Over the past decades, gene expression studies have demonstrated that key genes involved in neurodegenerative diseases are also expressed in skin tissues. Genes such as *APP*, *PSEN1*, *PPA2*, *PINK1*, *LRRK2*, *PLCB4*, *MAPT*, *SPAST*, and *SPG7* are prominent in this regard. Beyond gene expression, proteins related to neurodegenerative diseases—such as α-synuclein, TAU, PARKIN, and prion protein (PrP)—have been isolated from the skin of affected individuals, underscoring the skin’s capacity to mirror neural degeneration. This non-invasive window into neurodegenerative processes is further enhanced by advances in stem cell technology, which have allowed for the generation of human-induced pluripotent stem cells (iPSCs) from patient-derived fibroblasts. These iPSCs offer a valuable model for studying disease mechanisms and developing therapeutic approaches. This review conducts a comprehensive analysis of the literature from databases such as PubMed, Google Scholar, and ResearchGate, emphasizing the unique potential of the skin as a non-invasive biomarker for neurodegenerative diseases. It explores how the skin serves as a bridge between gene expression and disease pathology in both the skin and the CNS. By leveraging this biological connection, the skin emerges as a promising model for enhancing our understanding of neurodegenerative disorders and developing innovative strategies for early detection and treatment. However, significant limitations remain, requiring further validation to establish the specificity and sensitivity of these biomarkers.

## 1. Introduction

Neurodegenerative diseases of the CNS, including the brain, spinal cord, and retina, can affect individuals at various stages of life. These diseases range from childhood disorders to those more prevalent in older adults, such as Alzheimer’s Disease (AD) and Parkinson’s Disease (PD). While common conditions impact many, rarer diseases such as Huntington’s Disease (HD), amyotrophic lateral sclerosis (ALS), and frontotemporal dementia (FTD) also pose significant challenges. These disorders often have genetic, environmental, or complex origins and may lead to dementia, a condition projected to affect 150 million people globally by 2050, with an immense economic and social toll. As these diseases lead to progressive cognitive and motor decline, early diagnosis through non-invasive biomarkers is increasingly essential, especially with the aging global population driving a rise in prevalence [1].

Recent advances in biomarker research have identified skin fibroblasts as a promising tool for the early diagnosis of neurodegenerative diseases. These cells, easily obtained through a skin biopsy, share a common developmental origin with neural tissue (ectoderm), making them valuable for studying molecular and cellular processes in the brain [2]. Genetic mutations and protein abnormalities linked to diseases like Alzheimer’s (*APP*, *PSEN1*, *PSEN2*), Parkinson’s (*PINK1*, *LRRK2*), and Huntington’s (*HTT*) can be detected in fibroblasts, reflecting similar changes in brain pathology [3,4,5]. Additionally, protein biomarkers such as tau, α-synuclein, and Protein Kinase C Epsilon (PKCε), which are critical in neurodegenerative processes, can also be observed in skin fibroblasts, providing further insight into disease mechanisms [6,7,8].

Compared to blood samples, which can be affected by variables such as infections, medications, and nutritional status, skin fibroblasts provide more reliable and consistent results. Their uniform characteristics enable improved signal detection and reduce the variability commonly observed in blood-cell-based assays. However, skin fibroblast-based assays obtained through minimally invasive punch biopsies are not without limitations. One major drawback is the lag time between biopsy and test results, as the slow growth of fibroblasts in culture requires several weeks for completion [9].

Despite these challenges, the potential of a straightforward diagnostic skin test for AD has spurred ongoing innovations aimed at overcoming these technical hurdles. As a result, the development of skin fibroblast-based assays for AD remains a highly active area of research. Furthermore, the high diagnostic accuracy of skin prion Real-Time Quaking-Induced Conversion (RT-QuIC) demonstrates its value as either a complement to cerebrospinal fluid (CSF) analysis or as an alternative for patients unable to undergo lumbar puncture [10].

Although the slow growth of fibroblasts remains a challenge, continuous advancements in research are focused on improving the efficiency of these assays. Such efforts are positioning skin fibroblast-based tests as a promising tool for diagnosing and monitoring neurodegenerative diseases [9].

The advent of cell reprogramming technology has further expanded the potential of skin fibroblasts in neurodegenerative disease research. By reprogramming fibroblasts into induced pluripotent stem cells (iPSCs), scientists can produce disease-specific neurons in the laboratory, enabling a deeper comprehension of disease processes and the development of therapeutic interventions [11]. This breakthrough, pioneered by Yamanaka in 2006 [12], has transformed the field by enabling the creation of personalized, disease-specific cell models [13]. iPSCs derived from patients with neurodegenerative diseases have shown key disease traits in vitro, such as amyloid-β (Aβ) accumulation in AD, tau tangles in progressive supranuclear palsy (PSP), Corticobasal Degeneration Disease (CBD), and FTD, and α-synuclein aggregation in PD. These models have also accelerated the discovery of new treatments.

This article explores the use of skin fibroblasts and iPSCs as biomarkers for several neurodegenerative diseases, including AD, PD, HD, Creutzfeldt–Jakob Disease, PSP, CBD, ALS, Primary Lateral Sclerosis, Hereditary Spastic Paraplegia, and FD, highlighting their potential in transforming early diagnosis and therapeutic development.

## 2. Alzheimer’s Disease

AD, the primary cause of dementia worldwide, is expected to see its prevalence triple by 2050, with estimates rising even further when the disease is defined using biological markers rather than clinical symptoms. The disease’s progression begins at the cellular level, marked by the accumulation of amyloid β, which promotes the spread of tau proteins in the brain. Genetic predisposition plays a crucial role, accounting for 60–80% of AD risk, with more than 40 genetic risk loci identified, including the strongly associated *APOE* alleles [14]. While diagnosis has traditionally depended on clinical evaluations and imaging, recent research suggests that non-invasive methods, like studying skin fibroblasts, could offer new insights into AD. By examining gene expression and protein activity in these easily obtainable cells, researchers are identifying patterns that reflect the disease’s progression in the brain [3].

### 2.1. Genes Linked to AD in the Skin: The Role of APP, PSEN1, and PPA2

#### *APP* and *PSEN1*: Familial AD and Early Detection

Current diagnostic tools for AD are unable to detect the condition until significant and irreversible brain damage has occurred. Genetic studies on early-onset familial AD with autosomal dominant inheritance have identified three key genes involved in the disease: amyloid precursor protein (APP) and presenilins 1 and 2 (PSEN1 and PSEN2). A global gene expression analysis performed on fibroblasts from 33 individuals, including mutation carriers and their non-mutated siblings, demonstrated that carriers of the *APP^SWE^*, *APP^ARC^*, and *PSEN1 H163Y* mutations shared a distinct gene expression profile compared to non-carriers. However, the direct utility of *APP* and *PSEN1* genes in fibroblasts as biomarkers for AD is limited. No quantitative differences in their expression levels were observed between mutation carriers and wild-type siblings, and fibroblasts, as peripheral tissues, may not fully reflect the brain’s pathological processes. Biomarker identification relies on broader gene expression signatures rather than these genes alone, complicating the diagnostic process. Mutation-specific differences in clinical and neuropathological phenotypes further challenge the generalization of findings across familial AD cases. While pre-symptomatic changes can be detected, the lack of specific alterations in *APP* and *PSEN1* expression limits their role as standalone early biomarkers [3].

The implications of these findings are significant. By analyzing gene expression profiles in skin fibroblasts obtained from minimally invasive biopsies, researchers identified a specific gene signature associated with AD mutations. This advancement raises the possibility of presymptomatic diagnosis, enabling detection of the disease decades before symptoms like memory loss and cognitive decline emerge. Early diagnosis is critical for implementing interventions that could delay symptom onset, improve disease management, and enhance the quality of life for individuals at risk of AD [3].

### 2.2. PPA2 Gene: A New Player in Alzheimer’s Diagnosis

The *PPA2* gene, encoding serine/threonine phosphatase 2A, plays a pivotal role in critical cellular processes, including the dephosphorylation of tau protein, which is central to AD pathology. By dephosphorylating tau, *PPA2* helps regulate tau protein tangles, a hallmark of AD. Zhao et al. [15]. employed advanced techniques such as real-time quantitative polymerase chain reaction (RT-PCR) to analyze *PPA2* gene expression in fibroblast cells from AD patients compared to age-matched controls. Their findings revealed abnormal *PPA2* expression patterns in AD fibroblasts, accompanied by diminished phosphatase activity. Notably, an increase in *PPA2* mRNA concentrations was observed in some cases, possibly as a compensatory response to reduced enzymatic activity during disease progression. Despite this increase in mRNA, the enzymatic functionality of the *PPA2* protein remained impaired, contributing to the dysregulated cellular processes characteristic of AD [15].

In advanced stages of the disease, as seen in postmortem AD brain tissue, *PPA2* mRNA levels decline, suggesting that compensatory mechanisms fail to overcome the protein’s dysfunction. While changes in *PPA2* expression in peripheral tissues like fibroblasts may not entirely reflect brain-specific molecular events, they provide valuable insights into the disease’s underlying mechanisms. This positions *PPA2* as a potential biomarker for early diagnosis and a therapeutic target [15].

However, several restraints exist in using *PPA2* gene expression in fibroblasts as an AD biomarker. Peripheral fibroblasts, while accessible, may not fully capture the molecular events occurring in the brain, such as tau hyperphosphorylation and neurofibrillary tangle formation. A key limitation is the observed discrepancy between elevated *PPA2* mRNA levels and reduced protein activity in AD fibroblasts, suggesting that increased mRNA reflects a compensatory mechanism rather than a direct indicator of disease pathology. Furthermore, AD fibroblasts exhibit impaired responses to stimulation, unlike normal fibroblasts, which dynamically increase *PPA2* mRNA levels. This limits its reliability as a functional biomarker. The extent to which peripheral findings parallel central nervous system-specific impairments remains unclear, emphasizing the need for further validation of *PPA2* as a biomarker for AD [15].

### 2.3. Protein Biomarkers in Skin: The Role of PKC and Amyloid

#### Protein Kinase C Epsilon (PKCε) as an Early Biomarker

Khan et al. [8] investigated the role of PKCε in AD progression and its potential as an early biomarker. Using immunohistochemistry, they measured PKCε and amyloid-β (Aβ) levels in hippocampal neurons from postmortem AD brain samples and analyzed skin fibroblasts from AD patients, non-AD dementia patients, and healthy controls with ELISA. The study revealed a significant reduction in PKCε levels in the hippocampus and temporal pole of AD brains, accompanied by elevated Aβ levels. Similarly, fibroblasts from AD patients showed reduced PKCε levels compared to controls and exhibited distinct AD-related changes in how Aβ-oligomers impacted PKCε activity. This underscores the impaired interaction between PKCε and Aβ in AD, reducing PKCε’s protective role against toxic Aβ accumulation [8].

While these findings highlight the potential of fibroblast PKCε levels as a non-invasive biomarker for AD, there are limitations. Fibroblasts, as peripheral tissues, may not fully reflect brain-specific pathology, such as tau hyperphosphorylation or neurofibrillary tangle formation. The variability in PKCε levels caused by sample handling and culture conditions adds challenges to reproducibility. Additionally, the complex regulation of PKCε—affected by proteasome pathways and interactions with toxic Aβ oligomers—complicates its role as a diagnostic marker. The study also noted that the relationship between PKCε and Aβ might not be consistent across all disease stages or patient populations, particularly given the small cohort size and the lack of differentiation between familial and sporadic AD [8].

Despite these challenges, this study, the first to measure PKCε levels in both brain tissue and skin fibroblasts, suggests that fibroblast PKCε levels could serve as a promising diagnostic tool, mirroring the brain’s pathological changes. Larger studies are needed to validate its diagnostic accuracy and explore its potential for identifying diverse AD subtypes [8].

### 2.4. Amyloid Protein, Not a Definite Biomarker for AD

The accumulation of amyloid in skin fibroblasts cannot be considered a definitive biomarker for AD, as it has been found in both individuals with dementia and healthy controls. A study by Soininen et al. [16], which analyzed skin biopsies from patients with probable AD, multi-infarct dementia (MID), and non-demented controls, revealed positive amyloid-β (Aβ) immunoreactivity across all groups and in various skin regions. Similarly, Heinonen et al. [17] observed that while Aβ immunopositivity was more common in sporadic AD patients, it was also present in non-AD individuals, indicating that Aβ alone may not be a reliable diagnostic marker for AD [17]. Additionally, mast cells in the skin and stomach of AD patients have been found to express both Aβ and tau protein, suggesting that the effects of amyloid pathology may extend beyond the brain to involve other organs. This finding aligns with the growing evidence that AD may be a multisystem disorder, broadening the understanding of its systemic impact. However, the observation of positive Aβ immunoreactivity in both AD and non-AD individuals limits its specificity as a standalone biomarker. Peripheral amyloid accumulation may indicate systemic changes rather than AD-specific pathology, further complicating its diagnostic utility. The widespread presence of amyloid in various tissues and conditions highlights the need for additional research to clarify its role in non-brain pathologies and its potential relevance to AD progression and diagnosis [18].

Khalil et al. [19] further reported that Aβ deposition could impact vascular function in the skin, and their findings suggest that early endothelial changes, detectable through skin testing, could serve as an early indicator of AD, particularly in those with mild cognitive impairment or early symptoms. These findings point to the potential of using non-invasive skin tests as a diagnostic tool for early-stage AD patients [19].

### 2.5. Tau Protein in AD and Other Tauopathies

This year, Wang et al. [6] investigated the presence of specific tau proteins in peripheral tissues of elderly individuals across various tauopathies, including AD, progressive supranuclear palsy (PSP), corticobasal degeneration (CBD), and Pick’s disease (PiD). Using a seed-amplification assay (SAA) and ultrasensitive RT-QuIC, the study evaluated the prion-like seeding activity of pathological tau in skin samples from cadavers with confirmed tauopathies, comparing the results to healthy controls. The findings demonstrated that skin prion-SAA exhibited high sensitivity (75–80%) and specificity (95–100%) in identifying tauopathies. Remarkably, amplified tau-seeding activity was also detected in skin biopsies from living AD and PSP patients. This suggests that misfolded tau seeding in the skin could serve as a non-invasive biomarker for AD, potentially correlating with disease progression based on Braak staging, which tracks brain tau pathology. This approach highlights the promise of detecting tau pathology in peripheral tissues, offering a novel diagnostic tool for AD and related tauopathies and shedding light on tissue-specific vulnerabilities [6].

Even so, several challenges remain. Variability in tau detection across studies, likely due to differences in antibodies, experimental protocols, and sample preparation, emphasizes the need for methodological standardization. While the RT-QuIC assay demonstrates excellent sensitivity and specificity, its diagnostic reliability requires validation in larger and more diverse patient cohorts. Furthermore, cross-seeding interactions between tau and α-synuclein in comorbid conditions, such as AD and PD, complicate the interpretation of results and warrant further investigation [6].

Another limitation arises from the structural heterogeneity of tau across tauopathies. Variations in proteinase K-resistant fragments and other structural features introduce complexities that need to be better understood. Although tau seeding activity correlates with disease progression, its utility in monitoring disease severity remains uncertain. Additionally, the specificity of tau as a biomarker is questioned, given its presence in some synucleinopathies like dementia with Lewy bodies but not in others, such as multiple system atrophy. Addressing these challenges through larger studies, methodological refinements, and a deeper understanding of tau’s role in peripheral tissues will be critical for establishing skin tau as a reliable diagnostic biomarker [6].

### 2.6. Other Diagnostic Biomarkers for Alzheimer’s Diseases Using Skin Fibroblasts as Diagnostic Tools

*Disrupted calcium homeostasis* has been observed in both peripheral tissues and the brain of AD patients. Previous studies identified a significant decrease in cytosolic free calcium levels in fibroblasts from individuals with aging and AD compared to age-matched controls [20]. Further investigations into drug treatments revealed impaired calcium transport within the mitochondria of fibroblasts from AD patients, making these cells more vulnerable to oxidative stress caused by *free radicals*. This heightened sensitivity to oxidative stress in AD fibroblast mitochondria is associated with significant alterations in calcium uptake. Yet, this increased susceptibility may stem from general mitochondrial dysfunction rather than being exclusive to AD pathology, potentially limiting its diagnostic specificity [21].

While antioxidants such as U-74500A and deferoxamine were shown to partially protect AD mitochondria from oxidative stress-induced changes in calcium uptake, these treatments failed to completely reverse the alterations. This indicates that oxidative damage in AD fibroblast mitochondria is more complex and less reversible than in controls, suggesting that mitochondrial dysfunction in AD may involve broader, more intricate mechanisms. These findings highlight the challenges of using disrupted mitochondrial calcium homeostasis as a reliable biomarker for AD. Further research is needed to refine this approach and determine its specificity to AD pathology [21].

Skin fibroblast aggregation is an emerging diagnostic approach that assesses the rate at which skin fibroblasts cluster together, reflecting intercellular adhesiveness, a property often altered in disease states [22]. In this method, aggregation is quantified by calculating the average area per number of aggregates (A/N) after 48 h of cell plating on a Matrigel matrix. Studies have shown that individuals with AD exhibit a higher aggregation rate as cell density increases, making this technique a promising biomarker for identifying AD. Notably, this method demonstrated a 92% correlation with other diagnostic tools, such as the AD-Index, which evaluates ERK1/2 pathway imbalances. Such cross-validation emphasizes the accuracy of fibroblast aggregation as a peripheral diagnostic tool for AD, highlighting its potential in early detection and therapeutic intervention [22].

Nevertheless, several limitations temper the utility of fibroblast aggregation rates as a biomarker for AD. Overlapping aggregation rates between AD patients and age-matched controls (ACs), particularly near diagnostic thresholds, create a “gray zone” that complicates accurate classification. The study’s small sample size of 38 cases limits the generalizability of the findings. Additionally, fibroblast aggregation rates are highly influenced by experimental conditions, such as cell density, passage number, and culture protocols, which can impact reproducibility. Age-related changes in fibroblast behavior also act as a confounding factor, as aging affects aggregation rates and may obscure disease-specific signals [22].

Although the assay demonstrated strong concordance with other biomarkers like the AD-Index and morphology assays, it often required a “majority rule” approach to improve diagnostic accuracy, highlighting its limitations as a standalone biomarker. Moreover, the reliance on advanced imaging and statistical modeling may restrict its feasibility for routine clinical use without specialized resources [22].

The *AD-Index*, which tracks imbalances in MAP Kinase Erk1/2 phosphorylation, has emerged as a highly sensitive and specific biomarker for distinguishing AD patients from healthy controls and those with other forms of dementia [23]. Early cognitive impairment in AD is closely linked to inflammatory signaling pathways [24], prompting researchers to monitor the phosphorylation of MAPKs Erk1 and Erk2. These kinases are activated by bradykinin (BK), an inflammatory molecule that stimulates the PKC pathway. PKC, in turn, regulates key cytokines, which are significantly deficient in both the brains and skin fibroblasts of AD patients, reflecting widespread cellular dysfunction. In this diagnostic method, BK is applied to human skin fibroblasts obtained via punch biopsies, and the phosphorylation levels of Erk1 and Erk2 are measured. The resulting ratios are used to calculate the AD-Index, which has demonstrated exceptionally high sensitivity (97%) and specificity (100%) in autopsy-confirmed cases, making it a reliable diagnostic tool for AD [23].

However, the use of the AD-Index as a biomarker for AD is subject to several boundaries related to methodology and practical application. The accuracy of the index depends on highly controlled experimental conditions, particularly the precise measurement of phosphorylated Erk1 and Erk2 (P-Erk1 and P-Erk2) in response to BK stimulation. Variability in cell culture and assay techniques across laboratories can significantly affect results. Additionally, the AD-Index shows an inverse correlation with disease duration, proving most effective in early-stage AD but less reliable as the disease progresses. Overlap with non-AD dementias and comorbid conditions, such as AD combined with Parkinson’s or Lewy body disease, limits its specificity as a standalone diagnostic tool [23].

Moreover, the method requires advanced techniques like Western blot analysis and precise quantification, which may not be feasible for routine clinical applications. While it has shown high concordance with clinical diagnoses and autopsy findings, the small sample sizes in studies, particularly for mixed and non-AD dementia cases, restrict its generalizability. Khan et al. [25] further emphasized the index’s inverse correlation with disease duration, featuring its greatest utility for early diagnosis. Despite these limitations, the AD-Index holds significant promise as a peripheral biomarker for early recognition and intervention in AD, particularly due to its non-invasive nature and ability to enhance diagnostic certainty during the initial stages of the disease [25].

For broader clinical implementation, however, further validation is necessary. Larger studies, refinement of methodologies, and standardization across laboratories are critical to overcoming variability and establishing the AD-Index as a robust and reliable biomarker for AD [25].

In 2022, Chirila et al. [26] introduced an innovative diagnostic approach for AD using *Morphometric Imaging (MI)* in a double-blind, all-comers trial involving skin fibroblasts obtained through punch biopsies. The fibroblasts were cultured on a 3-D Matrigel matrix, and the dynamics of their movement and aggregation were analyzed. The study revealed that AD fibroblast lines formed fewer but larger aggregates compared to non-AD lines. These distinct differences in aggregate morphology enabled precise measurement and quantification of aggregate size and number, providing a novel means of detecting AD pathology. The biomarker’s accuracy was validated against autopsy-confirmed AD cases, with additional studies demonstrating its reliability even in the presence of co-morbid neurodegenerative conditions. The MI assay’s ability to differentiate AD from other neurodegenerative diseases and its potential to stratify patients for therapeutic trials highlight its significant utility in research and treatment [26].

Despite its promise, the MI assay as an AD biomarker faces several restrictions. Its accuracy is highly dependent on tightly controlled experimental conditions, including cell density, passage number, and imaging time points, making it sensitive to variability during preparation and execution. The method requires advanced imaging systems and specialized software to analyze fibroblast aggregation dynamics, which may restrict its accessibility for routine clinical applications. Furthermore, the process is time-intensive, involving prolonged culturing and multiple imaging phases over 48 h, which poses challenges for scalability in larger populations [26].

While the assay demonstrated high sensitivity and specificity, the study’s small cohort of 73 samples limits the generalizability of the findings to more diverse populations. Additionally, borderline cases with aggregation values near diagnostic cutoffs present a risk of misclassification. These challenges feature the need for further validation, standardization of methodologies, and optimization of the MI assay to establish it as a reliable, scalable, and feasible biomarker for AD [26].

In 2023, a pilot study by Wu et al. [27] identified significant differences in key physiological and biomechanical skin parameters between AD patients and healthy controls. The study revealed that AD patients exhibited a less acidic skin pH, reduced elasticity, and increased hydration compared to controls. Specifically, AD patients had more neutral skin pH values and higher hydration levels but significantly lower skin elasticity. These changes suggest potential alterations in skin physiology associated with AD [27].

Additionally, the study highlighted a negative correlation between baseline microvascular tortuosity ratios in the skin and cognitive function, as measured by the Mini-Mental State Examination (MMSE). This finding suggests a strong link between vascular changes in the skin and cognitive decline. To validate this relationship, the effects of cholinesterase inhibitor therapy were evaluated over six months. Patients who responded positively to the treatment showed significant improvements in skin capillary tortuosity ratios, accompanied by better MMSE and Clinical Dementia Rating-Sum of the Boxes (CDR-SB) scores, compared to non-responders. These results emphasize the potential of skin capillary tortuosity as a marker for cognitive improvements and treatment efficacy in AD [27].

While promising, the use of skin as a biomarker for AD, has several confines. Physiological skin parameters such as pH, hydration, and elasticity are influenced by external factors, including skincare routines, environmental exposure, and general health, which may confound their specificity as indicators of AD. Measuring parameters like capillary tortuosity and elasticity requires advanced equipment, such as laser Doppler flowmeters and dynamic capillary microscopes, making this approach less accessible and scalable in routine clinical settings [27].

The small sample size of 29 AD patients and 12 healthy controls further limits the generalizability of the findings. Additionally, skin biomarkers may reflect systemic changes, such as vascular or inflammatory alterations, that are not exclusive to AD and may overlap with other conditions, reducing specificity. For example, differences in hydration levels were partially attributed to the quality of care provided by family members, highlighting the impact of external, non-disease-related factors [27].

Finally, the study relied on cross-sectional data, providing limited insights into the progression of these skin biomarkers over time or their long-term response to treatments. This underscores the need for longitudinal research to validate the utility of these biomarkers for tracking disease progression and therapeutic outcomes in AD [27].

Other specific dysfunctions observed in skin fibroblasts from AD patients, which may have diagnostic potential, have involved abnormalities in cholesterol processing [28], K + channels [29], and folate binding [30].

### 2.7. iPSCs

The use of skin fibroblasts as biomarkers for AD has gained prominence with advancements in iPSC technology. iPSCs enable researchers to generate neurons from human skin fibroblasts, providing a more accurate model of AD pathology by retaining physiological gene expression and exhibiting disease-relevant traits such as amyloid-β (Aβ) accumulation and cellular stress [31]. A study by Armijo et al. [31] reprogrammed skin fibroblasts from patients with familial AD (fAD), sporadic AD (sAD), and healthy controls into iPSCs, which were then differentiated into neurons. These neurons were subjected to Aβ toxicity assays to assess their sensitivity to Aβ oligomers. The study found that neurons derived from fAD patients carrying the PSEN1-A246E mutation exhibited significantly greater sensitivity to Aβ 1-42 oligomers compared to neurons from sAD patients and healthy controls. This highlights the potential of iPSC-derived neurons from skin fibroblasts as biomarkers for AD, offering deeper insights into molecular mechanisms and aiding in the identification of individuals at elevated risk of developing AD [31].

Although promising, the application of iPSCs as biomarkers for AD is accompanied by several curbs. One significant challenge lies in the use of non-physiological concentrations of Aβ oligomers in toxicity studies, which may not accurately reflect chronic exposure in vivo. Although fAD iPSC-derived neurons show increased susceptibility to Aβ toxicity, this may result from intrinsic cellular stress and the complex interactions between oxidative and endoplasmic reticulum stress remain poorly understood [31].

The study’s focus on a small number of individuals with specific genetic mutations, such as PSEN1-A246E, restricts the generalizability of its findings to other AD forms and broader populations. Additionally, the acute in vitro exposure of neurons to Aβ oligomers fails to replicate the progressive and chronic nature of AD pathology. The technical demands of generating and differentiating iPSCs, which require advanced expertise and significant resources, further limit their scalability for clinical applications [31].

Another challenge is the variability introduced by the dynamic behavior of Aβ aggregates during experiments, which complicates reproducibility. These limitations emphasize the need for more physiologically relevant models, larger patient cohorts, and methodological standardization to validate the use of iPSC-derived neurons as robust and clinically applicable biomarkers for AD [31].

Below, Table 1 presents a detailed comparison of biomarkers for AD identified in skin fibroblasts. This comparison outlines the methods of detection, sensitivity, specificity, and key findings associated with these biomarkers, offering valuable insights into their utility and potential applications in AD research.

## 3. Parkinson’s Disease

PD, the second most prevalent neurodegenerative disorder, is primarily identified by motor symptoms such as tremors, slowed movement, stiffness, and balance problems, which result from the loss of dopamine-producing neurons in the brain. Non-motor symptoms, like digestive and cardiovascular issues, often appear long before motor symptoms, making early diagnosis challenging. Despite advances in neurodegenerative research, improved neuroimaging, and genetic studies, PD diagnosis still relies heavily on observable motor symptoms, which typically emerge after 60–80% of dopaminergic neurons have been lost. Promising biomarker research is now exploring the skin due to its shared origin with neural tissue and easy accessibility for testing [33].

### 3.1. Genes Linked to Parkinson’s Disease in the Skin: The Role of PINK1 and LRRK2

#### *PINK1* and *LRRK2* Genes Work Together

The *PINK1* gene, essential for maintaining mitochondrial health, has been implicated in early-onset PD. Azkona et al. [4] examined fibroblasts from PD patients with specific *PINK1* mutations and found no immediate *m*itochondrial dysfunction. However, these mutated cells exhibited elevated levels of *LRRK2*, another gene associated with PD. Introducing normal *PINK1* into these cells significantly reduced *LRRK2* protein levels, revealing a previously unknown role of *PINK1* in regulating *LRRK2* expression. This suggests a functional interaction between *PINK1* and LRRK2 in PD pathogenesis, positioning *PINK1* as a potential biomarker for the diseases [4].

For genetic analysis, conventional PCR was used to examine *PINK1* variants, particularly in exons 6 to 8. RNA was extracted and transcribed into cDNA for quantitative RT-PCR, which confirmed abnormal *LRRK2* expression in *PINK1* mutant cells. Neurons derived from patient fibroblasts using iPSC technology also exhibited increased *LRRK2* levels, further linking *PINK1* mutations to *LRRK2* dysregulation. These findings highlight the potential of monitoring *PINK1* and *LRRK2* interactions to gain insight into PD and identify early diagnostic markers and therapeutic targets [4].

Nonetheless, the use of *PINK1* and *LRRK2* as biomarkers for PD exhibits several shortcomings. Although *PINK1* mutations are linked to mitochondrial dysfunction, the observed phenotypes—such as normal ATP levels but increased glycolytic rates—are subtle and inconsistent, reducing their robustness as biomarkers. The interaction between *PINK1* and *LRRK2* in processes like mitochondrial dynamics, vesicle trafficking, and apoptosis is complex and highly variable across different cell types, limiting the generalizability of these findings [4].

While changes in *LRRK2* expression were observed in both *PINK1* mutant fibroblasts and iPSC-derived neurons, these alterations were not sufficiently distinct to serve as standalone biomarkers. They were more pronounced only in later stages of neuronal differentiation, restricting their utility for early detection of PD. Additionally, discrepancies between findings in fibroblasts and neurons suggest that peripheral tissue analyses may not fully capture CNS-specific pathological processes [4].

The study’s small sample size, which included only two patients with *PINK1* mutations and one carrier in iPSC experiments, limits the statistical power and applicability of the results to a broader population. These limitations accentuate the need for larger cohort studies and more comprehensive analyses to validate *PINK1* and *LRRK2* as reliable biomarkers for PD [4].

### 3.2. Protein Biomarkers in the Skin: The Role of LRRK2 and A-Synuclein in PD

#### LRRK2 Protein Therapeutic Potential

Therapeutic interventions targeting mitochondrial dysfunction and associated pathways have shown promising effects on peripheral biomarkers such as skin fibroblasts, particularly in PD. For instance, treatment with the LRRK2 inhibitor LRRK2-in-1 in PD patient-derived fibroblasts demonstrated significant improvements in mitochondrial health. This included the restoration of mitochondrial network integrity, membrane potential, and normalization of fission and fusion protein levels, such as OPA1, Mfn2, and Dlp1. Additionally, the intervention reduced LRRK2 phosphorylation and enhanced mitochondrial-lysosome co-localization, suggesting improved mitophagy, a process critical for clearing damaged mitochondria. Markers of oxidative stress, such as elevated nitric oxide and superoxide levels under stress conditions, were also reduced. Furthermore, the treatment influenced Parkin dynamics, promoting the formation of “Parkin rings,” structures linked to enhanced mitochondrial repair and mitophagy [34].

In another example, the LRRK2 inhibitor MLi-2 showed the ability to modulate several translationally repressed proteins in fibroblasts from both sporadic and LRRK2-G2019S PD patients. Proteins such as ATG9A (autophagy-related protein), YTHDF3 (mRNA processing), EHD1 (endolysosomal sorting), ABHD5 (lipid metabolism), and AP2B1 (endocytic trafficking) were restored after treatment, highlighting improvements in autophagy, proteostasis, and cellular trafficking pathways. For instance, ATG9A, essential for autophagosome formation, was upregulated, indicating enhanced autophagy and protein clearance. These findings suggest that peripheral fibroblast markers could serve as accessible and reliable indicators for evaluating the efficacy of disease-modifying therapies [35].

Despite these advancements, using LRRK2 protein and related pathways as biomarkers for PD unveils several challenges. One key issue is the variability in mitochondrial stress phenotypes among sporadic PD patient-derived fibroblasts, which complicates generalizing findings across all PD cases. Additionally, the complexity of mitophagy and mitochondrial dynamic pathways differs between LRRK2 mutation carriers and sporadic cases, making interpretations less consistent. Many findings are driven by artificial stress models, such as valinomycin-induced mitochondrial dysfunction, which may not fully replicate the chronic nature of PD pathology in vivo [34].

The small sample sizes—13 sporadic PD and 5 LRRK2-G2019S cases in the MLi-2 study—further limit the generalizability of findings. Translational repression of proteins, rather than direct mRNA alterations, complicates the identification of consistent biomarkers. Variability in affected pathways, such as inconsistent changes in ATG9A levels between sporadic and LRRK2-G2019S cases, further reduces reliability. The reliance on advanced proteomic techniques like BONCAT and targeted proteomics increases cost and limits clinical accessibility. Moreover, many pathways impacted by LRRK2 inhibition, such as autophagy and endo-lysosomal sorting, are common to other neurodegenerative diseases, reducing the specificity of these biomarkers for PD. Finally, findings in fibroblasts may not fully translate to CNS pathology, raising questions about their relevance to neuronal mechanisms [35].

Therefore, therapeutic interventions targeting LRRK2 pathways in fibroblasts demonstrate potential for improving mitochondrial and cellular health, as well as for serving as biomarkers for evaluating treatment efficacy. However, variability in pathways, limited cohort sizes, and the need for advanced technologies highlight the necessity of larger studies and further validation to establish LRRK2-related pathways as reliable and specific biomarkers for PD. These efforts will help determine the translational relevance of peripheral fibroblast findings to CNS pathology and broader PD contexts [34,35].

### 3.3. A-Synuclein Protein, Not a Definite Biomarker

Recent research underlines the growing potential of skin biopsies as a valuable tool for detecting phosphorylated α-synuclein (αSynP), a key biomarker for diagnosing synucleinopathies such as PD, dementia with Lewy bodies (DLB), multiple system atrophy (MSA), and pure autonomic failure (PAF). The Synuclein-One Study [7], a large-scale, multicenter project, demonstrated that skin biopsies offer high diagnostic reliability, with sensitivity exceeding 80% and specificity approaching 100%. Therapies aimed at reducing α-synuclein aggregation or enhancing its clearance via autophagy are expected to alter αSynP levels in skin fibroblasts, providing a measurable indicator of treatment efficacy. Reductions in αSynP deposition could reflect improvements in underlying disease mechanisms, reinforcing its utility as a dynamic biomarker [7,36].

Using immunohistochemical analysis, monitoring αSynP levels in skin fibroblasts provides a non-invasive means of evaluating therapeutic interventions. For instance, therapies enhancing protein clearance or inhibiting α-synuclein aggregation could reduce αSynP seeding activity and deposition, correlating with clinical improvements. This approach offers a minimally invasive alternative to cerebrospinal fluid analysis via lumbar punctures, expanding accessibility and patient comfort [7,36].

Although the use of α-synuclein as a biomarker for PD is promising, it has several impediments. The sensitivity of αSynP detection varies widely across studies. For example, while some studies, such as the Synuclein Systemic Sampling (S4) study, reported sensitivity as low as 24%, others demonstrated up to 94% sensitivity using RT-QuIC and PMCA assays, highlighting inconsistencies influenced by sample preparation and assay variability. Moreover, αSynP detection is highly specific to synucleinopathies but struggles to differentiate between subtypes, such as PD, DLB, and MSA, limiting its diagnostic precision [36,37].

Technical challenges further complicate its application. Variability in detection efficiency across biopsy sites (e.g., abdominal versus posterior scalp) and reliance on advanced imaging techniques like confocal microscopy introduce inconsistencies. Additionally, small sample sizes in many studies limit the generalizability of findings. Correlations between αSynP levels and disease severity have been reported, but its ability to reliably detect preclinical or prodromal stages of PD remains uncertain without longitudinal data. The absence of genetic testing and the exclusion of mimicking conditions, such as progressive supranuclear palsy (PSP) and corticobasal degeneration (CBD), also limits the biomarker’s specificity [37].

A landmark multicenter study conducted by Gibbons et al. [37], which included 428 participants with clinically confirmed PD, DLB, MSA, or PAF, demonstrated the practicality of αSynP detection in skin biopsies. Sensitivity rates were impressive, with 92.7% for PD, 98.2% for MSA, 96.0% for DLB, and 100% for PAF, while the control group exhibited only a 3.3% false-positive rate. Furthermore, αSynP levels correlated with disease severity, suggesting that skin biopsies could be used not only for diagnosis but also for monitoring disease progression and therapeutic efficacy [37,38].

While it appears advantageous, αSynP detection in skin biopsies is not yet FDA-approved for clinical use. Standardization of biopsy protocols, larger studies with diverse cohorts, and validation against neuropathological findings are essential to optimize its utility. The development of reliable skin biomarkers could revolutionize the diagnosis of synucleinopathies, enabling earlier and more accurate identification, improving patient outcomes, and accelerating the development of targeted therapies [38].

In conclusion, skin biopsies for αSynP detection hold significant promise as a non-invasive diagnostic and monitoring tool for synucleinopathies. While challenges remain, ongoing advancements in methodology and validation bring the field closer to realizing the clinical potential of this approach [38].

Table 2 bellow, summarizes the findings of Gibbons et al. [37], showcasing the sensitivity, specificity, and positivity rates for phosphorylated α-synuclein (αSynP) detection in skin biopsies across various synucleinopathies, highlighting its potential as a diagnostic and monitoring tool.

### 3.4. Enzyme Biomarker in the Skin: The Role of GCase in PD

Glucocerebrosidase (GCase), a lysosomal enzyme encoded by the *GBA* gene, plays a pivotal role in glycosphingolipid metabolism and has emerged as a significant biomarker for PD. Reduced GCase activity, a hallmark of both idiopathic and genetic PD, underscores lysosomal dysfunction as a central feature of PD pathology. Studies demonstrate that fibroblasts from idiopathic PD patients, even those without GBA mutations, exhibit markedly diminished GCase activity, comparable to levels observed in genetic PD cases with mutations such as N370S. This reduction is linked to decreased levels of the LIMP2 receptor, which is essential for transporting GCase to lysosomes. Consequently, GCase accumulates in the endoplasmic reticulum (ER) rather than being properly localized to lysosomes, pointing to trafficking defects as a primary cause of lysosomal dysfunction in idiopathic PD [39].

Assessing GCase activity in skin fibroblasts offers a non-invasive means of evaluating lysosomal dysfunction in PD and exploring therapeutic strategies. The observed reduction in GCase activity in idiopathic PD fibroblasts is attributed to impaired trafficking rather than reduced enzyme production, as transcriptional and translational levels remain unchanged. This consistency makes fibroblast GCase activity a reliable marker for systemic lysosomal health. Additionally, parallels between deficits observed in fibroblasts and those in brain tissues strengthen the case for using GCase as a systemic biomarker. This approach supports applications in early PD detection and in monitoring treatments aimed at enhancing LIMP2 expression or boosting GCase activity to mitigate substrate accumulation [39].

Despite its potential, the use of GCase activity as a biomarker for PD comes with several boundaries. GCase activity in fibroblasts does not strongly correlate with disease onset or progression, reducing its sensitivity to the disease stage. Furthermore, similar lysosomal deficits and glycosphingolipid accumulation are observed in other neurodegenerative diseases, such as dementia with Lewy bodies (DLB), diminishing its specificity for PD. The study’s small cohort, including 15 healthy controls, 31 idiopathic PD patients, and 6 genetic PD patients, limits the robustness of the findings. Additionally, accurate measurement of GCase activity requires specialized enzymatic assays and controlled conditions, complicating routine clinical application. Unexplored mechanisms may also contribute to GCase deficits, as decreased LIMP2 levels do not fully explain the observed reduction. Variations in methodology and tissue-specific differences between fibroblasts and neuronal cells further limit the translatability of findings to CNS pathology. These challenges necessitate larger, standardized studies and deeper exploration of underlying mechanisms to validate GCase activity as a reliable PD biomarker [39].

Therapeutic interventions such as Ambroxol, enzyme replacement therapy (ERT), and substrate reduction therapy (SRT) offer promising avenues to modulate biomarkers in peripheral tissues, including fibroblasts, in GBA1-associated PD. Ambroxol, a small-molecule chaperone, enhances GCase levels and activity by promoting proper folding of mutant GCase proteins and facilitating their delivery to lysosomes. It also increases GBA1 mRNA expression and lysosomal enzyme activity (e.g., cathepsins) and reduces cellular stress pathways such as ER stress. While ERT faces challenges due to the blood–brain barrier, it can normalize lysosomal substrate levels in peripheral tissues. SRT inhibits glucosylceramide synthase (GCS) to reduce substrate accumulation, potentially improving biomarkers like glycosphingolipids in peripheral tissues [40].

These therapies are expected to normalize biomarkers such as GCase activity, reversing the hallmark deficits of GBA1 mutations. Substrate levels, including glucosylceramide (GlcCer) and glucosylsphingosine (GlcSph), should decrease, signaling improved metabolic balance. Enhanced lysosomal function and autophagy-related protein activity further indicate restored cellular homeostasis. These measurable changes in fibroblast biomarkers provide valuable tools for monitoring the efficacy of targeted therapies in clinical trials and for patient management [40].

Therefore, the assessment of GCase activity in skin fibroblasts represents a promising avenue for understanding lysosomal dysfunction in PD and evaluating the efficacy of therapeutic interventions. While limitations such as specificity and cohort size need to be addressed, advancements in therapeutic approaches like Ambroxol and SRT highlight the potential of peripheral biomarkers in guiding disease management [39,40].

### 3.5. iPSCs

The application of iPSC technology to derive dopaminergic neurons (DAn) from skin fibroblasts has significantly advanced their use as biomarkers for PD. In a recent study, iPSCs were generated from skin fibroblasts obtained from individuals with idiopathic PD (ID-PD), familial PD associated with the G2019S mutation in the LRRK2 gene, and healthy, age- and sex-matched controls. When differentiated into DAn, neurons derived from PD patients displayed disease-specific features, including reduced neurite outgrowth, diminished arborization, and the accumulation of autophagic vacuoles. These abnormalities were absent in neurons from healthy controls, demonstrating that iPSC-derived neurons effectively model the genetic and cellular complexity of PD. Further investigation revealed that disrupted autophagy, particularly impaired autophagosome clearance, contributed significantly to these neuronal defects. Enhancing autophagy or inhibiting lysosomal activity exacerbated the morphological abnormalities, underscoring the critical role of autophagic dysfunction in PD progression. These findings position iPSC-derived neurons as a valuable platform for studying idiopathic and familial forms of PD. They not only capture the genetic characteristics of the disease but also offer a practical system for exploring disease mechanisms and testing potential therapeutic strategies [41].

Nevertheless, the use of iPSC-derived neurons as biomarkers for PD comes across several challenges. Disease-related phenotypes, such as reduced neurite length and increased apoptosis, typically emerge only after prolonged cultures of over 2.5 months. While these long-term cultures attempt to mimic aging, they fall short of replicating the progressive and dynamic nature of PD neurodegeneration. Moreover, observed phenotypes may be influenced by in vitro stress conditions rather than representing true disease pathology, complicating the interpretation of results. Inconsistencies across studies further challenge the reliability of this model. Some studies have failed to observe PD-related changes in iPSC-derived neurons without the application of external stressors, indicating variability in replicating PD pathology. The generation and differentiation of iPSCs into DAn require advanced technical expertise, lengthy culture periods, and rigorous protocols, limiting their scalability for routine clinical use. Additionally, the study involved a small cohort of seven idiopathic PD and four LRRK2-associated PD patients, which restricts the generalizability of the findings to the broader PD population. These challenges stress the importance of refinement and standardization of iPSC methodologies to enhance their reliability and applicability as PD biomarkers [41].

iPSC-derived neurons from skin fibroblasts offer a promising model for studying the genetic and cellular underpinnings of PD. They provide insights into key disease processes, such as autophagic dysfunction, and serve as a platform for testing therapeutic interventions. However, limitations related to prolonged culture requirements, variability in disease replication, and the technical demands of the methodology highlight the need for further standardization and larger studies to establish iPSCs [41].

## 4. Huntington’s Disease

HD is a genetic neurodegenerative disorder resulting from the expansion of polyglutamine (polyQ) in the huntingtin protein (Htt), which leads to mitochondrial dysfunction and disruption of the ubiquitin–proteasome system (UPS) in neurons [42]. Although the disease’s effects outside the nervous system are not fully understood, HD is primarily characterized by motor, cognitive, and behavioral impairments due to a CAG repeat expansion in the HTT gene. The degeneration of medium spiny neurons in the striatum is a hallmark characteristic of the disease and explains many of its clinical symptoms. Early signs preceding neuronal loss point to abnormal striatal neuron function in the initial stages. Current treatment options focus on symptom management and supportive care, but emerging research on skin biomarkers holds promise for improving early diagnosis and developing new therapies [43].

### 4.1. Genes Linked to HD in the Skin: The Role of PLCB4, UBE2D3, APC, and ROCK1

#### *PLCB4*, *UBE2D3*, *APC*, and *ROCK1* Genes Upregulated in HD

Research highlights the potential of skin-derived fibroblasts as biomarkers for studying gene expression in HD, offering a practical alternative to examining CNS tissues. The mutated huntingtin gene (*mHTT*), which causes HD, is expressed systemically, making fibroblasts an accessible and valuable tool for investigating peripheral molecular changes. Unlike post-mortem brain tissues, which often suffer from RNA degradation, fibroblasts are easier to collect and maintain. Although gene expression in fibroblasts can be influenced by culture conditions, these cells remain a reliable platform for HD research outside the CNS [5].

A study identified four key genes—*PLCB4*, *UBE2D3*, *APC*, and *ROCK1*—that were significantly upregulated in HD patients compared to healthy controls. These genes are involved in essential cellular processes, including RNA processing, cellular signaling, and cytoskeletal organization. Their upregulation mirrors dysfunctions observed in HD-affected neurons, suggesting that fibroblasts experience molecular disruptions similar to those occurring in the nervous system. This finding accentuates the value of fibroblasts in studying the systemic effects of HD and provides insights into molecular mechanisms underlying the disease. The research also opens avenues for developing diagnostic tools and therapeutic strategies, positioning fibroblasts as a critical resource for advancing HD treatment [5].

While *PLCB4*, *UBE2D3*, *APC*, and *ROCK1* genes hold potential as biomarkers for HD, several gaps must be addressed to validate their utility. The findings remain preliminary, with differential gene expression requiring protein-level validation to confirm functional relevance. The study’s small cohort size limits the generalizability of the results to broader HD populations. Moreover, gene expression in fibroblasts is susceptible to external factors such as culture conditions, growth phases, and passage numbers, potentially introducing variability and reducing reproducibility.

Additionally, while fibroblasts provide an accessible peripheral model for HD, they may not fully recapitulate the molecular mechanisms specific to neuronal cells directly affected by the disease. This limits their utility as standalone biomarkers. To establish these genes as reliable biomarkers for HD, larger studies and integrated datasets combining fibroblast and CNS-derived data are essential [5].

Skin-derived fibroblasts offer a promising platform for HD research, capturing peripheral molecular changes associated with the disease. The upregulation of *PLCB4*, *UBE2D3*, *APC*, and *ROCK1* in HD fibroblasts highlights their potential for studying systemic effects and developing diagnostic tools. However, challenges such as small cohort sizes, external variability, and the need for protein-level validation emphasize the need for further research [5].

### 4.2. Protein Biomarkers in Skin: The Role of Parkin in HD

#### Parkin Protein: Possible Protective Role

Parkin protein, essential for maintaining mitochondrial health and regulating proteasome activity, has gained prominence in the study of juvenile HD. In skin fibroblasts derived from juvenile HD patients, researchers observed mitochondrial abnormalities, including increased cell size, elevated reactive oxygen species (ROS), and disruptions in mitochondrial fusion and fission. Despite these mitochondrial challenges, the fibroblasts remained viable. Interestingly, parkin expression was elevated in these cells and appeared to play a protective role by mitigating cellular and mitochondrial damage. This protective function was achieved through enhanced proteasome activity, which facilitated the rapid degradation of ubiquitin–proteasome system (UPS) targets, such as the mitochondrial fusion protein Mfn1, thereby preserving cellular function. These findings suggest that parkin acts as a safeguard, maintaining cell stability and function under HD-related stress [42].

Therapeutic interventions targeting proteasome activity and mitochondrial quality control hold significant potential for improving fibroblast function in juvenile HD patients. Proteasome activity, naturally elevated in HD fibroblasts as an adaptive response to mHTT protein, could be further augmented with therapies such as proteasome activators or molecular chaperones. These treatments would enhance the clearance of misfolded proteins and reduce toxic protein aggregates, alleviating cellular stress. Similarly, therapies designed to optimize mitophagy could regulate parkin levels and improve mitochondrial quality control. Enhanced parkin activity would restore mitochondrial networks, promote better energy balance, and ensure healthier cellular function [42].

Therapies addressing oxidative stress and mitochondrial dynamics are also expected to significantly impact fibroblast biomarkers. Antioxidants could reduce ROS levels and stabilize mitochondrial membrane potential, minimizing oxidative damage. Interventions aimed at correcting imbalances in mitochondrial fusion and fission—such as those targeting reduced Mfn1 levels—would prevent excessive mitochondrial fragmentation and restore proper mitochondrial function. Together, these strategies would normalize key fibroblast biomarkers, including proteasome activity, oxidative stress markers, and mitochondrial dynamics, providing a non-invasive method for evaluating treatment effectiveness in HD while improving overall cellular health [42].

The use of parkin protein as a biomarker for HD presents several flaws that affect its specificity and broader applicability. While parkin levels are elevated in juvenile HD fibroblasts, it remains unclear whether this represents a disease-specific adaptation or a generalized cellular response to stress, reducing its specificity as an HD biomarker. Its role in HD fibroblasts appears to be compensatory, linked to proteasomal degradation of mitochondrial proteins like Mfn1, making it an indirect indicator of pathology. Additionally, parkin expression is closely tied to proteasome activity, which may vary under different experimental conditions or disease stages, potentially affecting its reliability [42].

The mechanism by which parkin modulates mitochondrial dynamics is complex and may not fully reflect mitochondrial dysfunction in all HD cases, limiting its generalizability. Furthermore, the study focuses exclusively on juvenile HD fibroblasts, which may respond differently from adult-onset HD cases, raising questions about the broader relevance of these findings. Lastly, as these results are based on fibroblast models, further validation in neuronal cells or other systems is necessary to confirm the systemic relevance of parkin as a biomarker for HD [42].

Parkin protein appears to play a crucial protective role in juvenile HD fibroblasts, mitigating mitochondrial dysfunction and cellular stress. This highlights its potential as a therapeutic target and a biomarker for monitoring treatment efficacy. However, limitations related to specificity, generalizability, and experimental variability underscore the need for further research [42].

### 4.3. iPSCs

The use of skin fibroblasts as biomarkers for HD has advanced significantly with the development of iPSC technology [44]. In 2008, researchers successfully reprogrammed fibroblasts from HD patients into iPSCs, offering a novel approach to model the disease [45]. However, early attempts to create HD models using iPSC-derived neurons faced limitations, as these neurons often failed to exhibit the hallmark protein aggregates or neuronal death associated with HD. This shortcoming was largely attributed to the erasure of cellular age signatures during reprogramming, which are critical for modeling late-onset HD phenotypes. Additionally, the manifestation of HD-specific features in iPSC-derived neurons required the application of external stressors, such as oxidative stress or trophic factor withdrawal, and took 6–8 months in culture, making the process time-intensive and less practical for routine biomarker applications. Furthermore, the proteostasis machinery in iPSCs is typically more efficient than in aged cells, potentially masking key HD phenotypes such as mHTT aggregation [46].

Recent advances have addressed these limitations by employing microRNA-based direct neuronal conversion, which transforms HD patient fibroblasts directly into medium spiny neurons (MSNs) while preserving the cells’ age-associated characteristics. This approach has led to the generation of neurons that consistently exhibit critical HD features, including mHTT aggregates, DNA damage, mitochondrial dysfunction, and spontaneous neuronal degeneration, without requiring external stressors. The retention of age-related signatures in the converted neurons has been particularly valuable for modeling the role of aging in HD pathology, especially in late-onset cases. These improvements have enhanced the accuracy of HD models, enabling researchers to study disease progression more effectively and facilitating the development of targeted therapies [46].

While iPSC experience remains a valuable research tool, its challenges in modeling age-related phenotypes and inconsistent expression of HD-specific traits highlight the advantages of direct neuronal conversion. The latter provides a more reliable and accurate representation of HD pathology by capturing age-related molecular and cellular changes. However, the lack of functional validation across diverse patient cohorts underscores the need for broader studies to standardize findings. These developments suggest that direct neuronal conversion offers a more practical and promising alternative for modeling HD and developing biomarkers, advancing our understanding of the disease and its potential treatments [46].

Bellow, Table 3 compares early and recent iPSC models for HD as highlighted in recent research. [46]. It highlights the advancements made through microRNA-based direct neuronal conversion, which retains age-related characteristics and consistently exhibits hallmark HD features, unlike earlier iPSC-derived neuron models that lacked age-related relevance and key disease phenotypes.

## 5. Sporadic Creutzfeldt–Jakob Disease

sCJD is a swiftly progressing and lethal neuropsychiatric disease caused by the buildup of misfolded prion proteins (PrPSc) in the brain. It is the most prevalent form of human prion disease, accounting for approximately 90% of cases, with an incidence rate of 1.5 to 2.0 cases per million person-years. The clinical presentation of sCJD can vary significantly, with different phenotypes influenced by factors such as the Methionine/Valine (M/V) polymorphism at codon 129 of the PRNP gene and the molecular subtype of PrPSc. A definitive diagnosis requires confirmation through neuropathological examination, but typical symptoms include rapidly progressive dementia, cerebellar ataxia, and myoclonus, alongside pathological hallmarks such as spongiform degeneration, neuronal loss, and astrocytosis in the brain [47,48]

Due to the variability in symptoms, the use of reliable biomarkers is essential for accurate diagnosis. In 1998, the World Health Organization (WHO) established diagnostic criteria for sCJD, incorporating a range of clinical features, electroencephalography (EEG), and the detection of 14-3-3 proteins in CSF [47].

### 5.1. Protein Biomarkers in Skin: The Role of PrPSc and Prion Seeding Activity in CJD

#### PrPSc Protein

A study investigated prion seeding activity and infectivity in skin samples from individuals with sporadic Creutzfeldt–Jakob disease (sCJD). Skin samples were collected from 21 sCJD patients, 2 variant CJD (vCJD) patients, and 15 non-CJD controls. PrPSc, the misfolded prion protein associated with prion diseases, was analyzed using Western blotting and RT-QuIC assays. While Western blotting detected PrPSc in only a few cases, RT-QuIC successfully identified prion seeding activity in the skin of all CJD patients and none of the non-CJD controls. Prion infectivity was confirmed by inoculating skin homogenates from two sCJD patients into humanized transgenic mice, all of which developed prion disease. Although prion seeding activity in the skin was significantly lower than in brain tissue, these findings demonstrate that sCJD patient skin contains both prion seeding activity and infectivity. This raises concerns about potential prion transmission through skin contact or medical procedures involving the skin [48].

The discovery of prion seeding activity and infectivity in the skin also highlights the potential for using skin-derived fibroblasts as biomarkers for sCJD. While prions were consistently detected in the skin samples of sCJD patients and absent in non-CJD controls, it remains unclear at what stage of the disease prions appear in the skin. Additionally, the significantly lower prion seeding activity in skin compared to brain tissue poses challenges for its diagnostic utility. The study does not confirm whether RT-QuIC testing of skin samples could serve as a practical diagnostic tool, and further research is needed to explore the diagnostic potential of skin-derived fibroblasts and assess the transmission risks during skin-related medical procedures [48].

The use of PrPSc as a biomarker for sCJD faces several limitations that impact its practicality and reliability. Prion seeding activity in the skin is approximately 1000-fold lower than in brain tissue, limiting sensitivity, particularly in early or less advanced stages of the disease. Prion distribution in skin tissue is variable, with no single location consistently yielding positive results, complicating the standardization of sampling protocols. Highly sensitive assays such as RT-QuIC are required for detection, but these are resource-intensive and not widely available in clinical settings, while conventional methods like Western blotting fail to detect PrPSc in most skin samples. Furthermore, the timing and appearance of PrPSc in the skin during disease progression remain poorly understood, restricting its use for early diagnosis. Although infectivity in human skin was demonstrated in transgenic mouse models, the study does not comprehensively address the transmission risk in humans or the implications for non-CNS surgical procedures. Finally, the small study size limits the generalizability of the findings, emphasizing the need for larger studies with refined methodologies to validate the use of skin as a diagnostic or research tool for prion diseases [48].

### 5.2. Prion Seeding Activity and Infectivity

The detection of prions in skin punch biopsies from CJD patients using RT-QuIC has demonstrated promising diagnostic accuracy. This method offers a minimally invasive alternative or complement to CSF analysis, particularly for patients unable to undergo a lumbar puncture. Mammana et al. [10] showed that prion-seeding activity could be reliably identified in small tissue samples from skin biopsies, with the cervical area yielding the most consistent positive results. The findings also suggest that prion replication extends beyond the CNS, with prion-seeding activity in skin varying by strain type, such as M1 and V2. Subtypes VV2 and MV2K exhibited higher prion-seeding activity in skin samples compared to MM1. Additionally, prion-seeding activity appears to increase as the disease progresses, indicating a potential spread from the CNS to peripheral tissues. While skin punch biopsies represent a valuable diagnostic alternative for detecting prion-seeding activity, further research is required to evaluate their implications for infectivity and transmission risks [10].

The use of prion-seeding activity and infectivity as biomarkers for sCJD presents several limitations that affect their reliability and applicability. Detection rates vary across sCJD subtypes, with lower sensitivity observed in cases such as sCJDMM1 compared to VV2 and MV2K, limiting the biomarker’s effectiveness across all forms of the disease. Moreover, prion-seeding activity in the skin is generally less sensitive than CSF analysis, particularly in subtypes with reduced peripheral deposition of PrPSc. As prion-seeding activity increases with disease progression, its utility for early diagnosis is constrained, as levels may be undetectable in the initial stages of the disease. Biopsy location also impacts detection rates, with cervical skin yielding better results than thigh samples, complicating the standardization of sampling protocols [10].

The limited number of cases tested outside the MM1 group reduces the generalizability of the findings, emphasizing the need for larger and more diverse cohorts. Additionally, the reliance on RT-QuIC technology, which requires specialized equipment and technical expertise, limits accessibility for routine clinical use. While experimental studies have demonstrated prion infectivity in skin samples, the implications for human transmission through medical procedures involving the skin remain unclear, requiring further investigation [10].

In conclusion, while the detection of prion-seeding activity in skin punch biopsies offers a promising diagnostic tool for sCJD, its current limitations highlight the need for larger studies, methodological refinements, and comprehensive assessments of transmission risks. Such efforts are essential to validate prion-seeding activity and infectivity as robust and reliable biomarkers for sCJD [10].

## 6. Progressive Supranuclear Palsy (PSP) and Corticobasal Degeneration (CBD)

PSP and CBD are both neurodegenerative disorders classified as tauopathies, marked by the build-up of abnormal tau protein, specifically the 4-repeat tau isoforms. Although the brain regions affected by PSP and CBD overlap, differences exist in the patterns of neuronal damage, the characteristics of glial lesions, and the prevalence of accompanying pathologies. Both conditions exhibit a wide range of symptoms affecting movement and cognition. Early diagnosis can be challenging, especially for CBD’s corticobasal syndrome, while PSP’s Richardson syndrome is more commonly recognized. Understanding their variability may help improve diagnostic precision [49].

### 6.1. Gene Linked to PSP and CBD in Skin: The Role of MAPT

#### *MAPT* Gene Distinguishes Tauopathies from α-Synucleinopathies

The *MAPT gene*, which encodes the tau protein, has been explored as a potential biomarker for distinguishing tauopathies, such as PSP and CBD, from other neurodegenerative diseases, including PD and multiple system atrophy (MSA). Recent studies employed real-time PCR to analyze *MAPT gene* expression in fibroblasts derived from skin biopsies, offering a non-invasive method to measure tau levels in peripheral tissues. By examining mRNA levels of specific tau isoforms, particularly the 2N and 4R variants, researchers identified distinct differences in gene expression patterns. Patients with tauopathies such as PSP and CBD exhibited higher expression of these MAPT isoforms compared to those with other neurodegenerative conditions, indicating a stronger involvement of tau pathology in these diseases [50].

The study’s findings highlight the ability of using skin fibroblasts, combined with real-time PCR, as a reliable biomarker for diagnosing tauopathies. Such approaches could significantly enhance early diagnosis and differentiation of neurodegenerative conditions, allowing for more timely and personalized treatment strategies. This non-invasive technique provides an accessible and practical alternative for identifying tau pathology in living patients, addressing a critical need in clinical practice [50].

Still, there are several constraints to using the *MAPT gene* as a biomarker for PSP and CBD. The small sample size of the study reduces the generalizability of the findings, and the absence of post-mortem pathological confirmation introduces potential inaccuracies in clinical diagnoses. The study focused solely on soluble tau protein isoforms, such as 2N and 4R, without assessing insoluble aggregates that are more directly associated with tau pathology. Additionally, the analysis excluded other post-translational modifications, such as acetylation and ubiquitination, which may play a significant role in tau-related neurodegeneration [50].

The use of a custom ELISA for tau quantification further raises concerns about reproducibility, as standardized commercial assays are not yet widely available. Moreover, the study did not detect the Big-tau isoform, which predominates in larger peripheral nerve fibers, potentially limiting the understanding of tau’s broader role in PSP and CBD pathology. These gaps highlight the need for more comprehensive biochemical analyses and larger cohort studies to validate MAPT gene products as reliable biomarkers [50].

As a final point, the investigation into MAPT gene expression in fibroblasts offers a promising avenue for diagnosing tauopathies and distinguishing them from other neurodegenerative diseases. However, to fully establish its clinical utility, future research must address current limitations through expanded sample sizes, post-mortem validation, and a more detailed assessment of tau isoforms and post-translational modifications. These efforts will help refine MAPT-based biomarkers, enhancing their reliability and specificity for clinical applications [50].

### 6.2. Protein Biomarker in Skin: The Role of Tau in PSP and CBD

#### Tau Protein 2N4R Isoform in PSP and CBD

Tau protein, a critical stabilizer of microtubules in neurons, has garnered attention as a potential biomarker for neurodegenerative diseases, particularly tauopathies such as PSP and CBD. In the study by Vacchi et al. [50], different tau isoforms produced through alternative splicing of the MAPT gene were analyzed using Western blotting. Isoforms such as 0N4R, 1N3R, and 2N4R, which are associated with tau accumulation in tauopathies, were identified. The findings revealed that the 2N4R isoform was significantly more abundant in patients with PSP and CBD compared to those with PD and MSA. Using fibroblasts from skin tissue as a source of tau protein, combined with Western blotting, provides a promising non-invasive method for distinguishing tauopathies from other neurodegenerative disorders. This technique holds the potential for improving early and accurate diagnoses, thereby facilitating timely and targeted interventions for patients with tau-related disorders [50].

The use of the tau protein 2N4R isoform as a biomarker for PSP and CBD, however, is not without drawbacks. The study included only a small group of patients with PSP and CBD, which may reduce the statistical power and generalizability of the findings. Furthermore, the analysis focused exclusively on soluble tau isoforms, overlooking insoluble aggregates that are more directly associated with tau pathology. Methodological constraints, such as reliance on a custom ELISA for tau quantification, raise concerns about reproducibility across different laboratories and studies [50].

Additionally, the study identified two tau isoforms (55 kDa and 70 kDa) in skin samples, distinct from the six isoforms typically observed in the human adult brain. This discrepancy raises questions about the extent to which skin-derived tau biomarkers reflect CNS pathology. The absence of Big-tau, an isoform associated with large peripheral nerve fibers, further highlights potential limitations in using skin as a model for peripheral tauopathy analysis. While increased levels of 2N and 4R tau isoforms were observed in PSP and CBD patients, the lack of post-mortem pathological confirmation and incomplete evaluation of post-translational modifications, such as acetylation and ubiquitination, limit the broader clinical applicability of these findings [50].

In summary, tau protein isoform analysis in skin fibroblasts offers a promising, non-invasive avenue for diagnosing tauopathies and distinguishing them from other neurodegenerative diseases. However, to establish the diagnostic utility of the 2N4R isoform and related tau biomarkers, larger patient cohorts, expanded analyses of isoforms and post-translational modifications, and standardized methodologies are essential. These efforts will enhance the reliability and specificity of tau-based biomarkers, paving the way for their integration into clinical practice for the management of tau-related disorders [50].

## 7. Amyotrophic Lateral Sclerosis (ALS) and Primary Lateral Sclerosis (PLS)

ALS is a neurodegenerative disorder that leads to the progressive loss of both upper and lower motor neurons, affecting movement control via the brain, brainstem, and spinal cord. Symptoms often start in the limbs or bulbar muscles, though respiratory muscles can be affected in rare cases. Most people with ALS survive 2–3 years after symptom onset, with a minority living beyond 10 years. ALS is the most prevalent form of motor neuron disease (MND), which also includes conditions like PLS. PLS involves only upper motor neuron degeneration for at least four years and typically follows a slower, less aggressive progression, with survival extending up to 17 years. Though PLS shares some clinical and pathological features with ALS, it progresses more gradually and is less understood due to its rarity. Some PLS cases can later develop lower motor neuron involvement, signaling a more rapid disease course [51]. In 2015, Raman et al. [51] conducted a study to investigate gene expression in fibroblasts from patients with ALS and PLS. The study aimed to assess whether fibroblasts could serve as cellular models for these motor neuron diseases, particularly in sporadic ALS (SALS) and PLS, where genetic modeling is more difficult [51].

### 7.1. Genes Linked to ALS and PLS in the Skin

#### Several Genes

Genes associated with ALS and PLS have been investigated in skin fibroblasts, offering a promising peripheral model for studying these motor neuron diseases. Through microarray analysis, Raman et al. identified several genes with altered expression in SALS (sporadic ALS) and PLS fibroblasts, particularly in pathways related to RNA processing and hypoxia response. These findings were validated using quantitative PCR and functional assays and reflected similar disruptions observed in the CNS of ALS patients. Notably, SALS fibroblasts exhibited broader transcriptional changes compared to PLS fibroblasts, with pronounced alterations in genes linked to energy metabolism and stress responses. These included reduced expression of genes involved in microRNA production and hypoxia response, leading to impaired cellular respiration and RNA metabolism, mirroring neuronal disruptions seen in ALS [51].

Both SALS and PLS fibroblasts display metabolic changes, including alterations in glycolysis and lipid metabolism. An adaptive increase in fatty acid oxidation was observed, suggesting a compensatory mechanism to meet energy demands. These findings indicate that fibroblasts not only reflect certain CNS disruptions but also provide valuable insights into the systemic aspects of ALS and PLS pathology. As genetic models for these diseases can be challenging to develop, fibroblasts present an accessible alternative for exploring disease mechanisms and identifying therapeutic targets [51].

Although showing promise, fibroblast-derived biomarkers for ALS and PLS encounter considerable challenges that impact their utility. Transcriptional heterogeneity is a major obstacle, as SALS fibroblasts exhibit more extensive transcriptional changes compared to PLS, complicating the identification of consistent biomarkers across patient populations. While some dysregulated processes in fibroblasts parallel those observed in the CNS, many CNS-specific mechanisms are absent, limiting the comprehensiveness of these biomarkers. Additionally, overlap in differentially expressed genes between ALS and PLS fibroblasts reduces their specificity for distinguishing between the two diseases [51].

Functional discrepancies further complicate biomarker development. For example, impaired hypoxia responses and reduced glycolytic adaptation in SALS fibroblasts may not fully align with the mechanisms driving neuronal degeneration. Dysregulated RNA processing pathways, highlighted by significant reductions in microRNA levels, present additional challenges due to variability in miRNA profiles across patients. Compensatory mechanisms in fibroblasts, such as changes in stress-response gene expression, may obscure the interpretation of biomarker data [51].

Thus, while skin fibroblasts serve as a convenient and accessible peripheral model for ALS and PLS, their limitations necessitate careful consideration when translating findings into reliable clinical biomarkers [51].

### 7.2. iPSCs

The use of skin fibroblasts as biomarkers for ALS has gained significant importance with advancements in iPSC technology. In a study by Bhinge et al. [52], researchers generated motor neurons from iPSCs derived from ALS patients’ fibroblasts and compared them to motor neurons corrected using CRISPR technology. This approach allowed for a detailed exploration of why motor neurons are particularly vulnerable in ALS. The study identified the transcription factor JUN, a component of the AP1 complex, as being expressed at significantly higher levels in motor neurons compared to other neuronal types, suggesting JUN plays a critical role in motor neuron health. By comparing diseased and gene-corrected neurons, the researchers linked JUN overexpression to the SOD1 mutation, offering insights into the mutation’s role in ALS progression [52,53].

The research further revealed that key signaling pathways, including ERK and JNK, are dysregulated in ALS motor neurons, contributing to neurodegeneration in individuals carrying the SOD1 mutation. JUN, as a target of these pathways, was found to be markedly elevated in motor neurons, which may explain their heightened vulnerability to degeneration. Similar disruptions were observed in motor neurons with other ALS-associated mutations, such as FUS, affecting pathways like p38 and ERK. These findings indicate that different ALS-related mutations converge on common cellular processes, many of which are amenable to drug targeting. This emphasizes the potential of iPSC-derived motor neurons from fibroblasts as a valuable model for understanding ALS and for developing targeted therapeutic strategies [52,53].

The use of iPSC-derived neurons as biomarkers for ALS and PLS is hindered by several challenges, including phenotypic variability caused by genetic differences between individual iPSC lines, making it difficult to identify consistent biomarkers. Additionally, iPSC-derived neurons often resemble immature or “fetal-like” cells, limiting their ability to fully replicate the mature neuronal states and pathologies observed in ALS and PLS patients. While these models successfully highlight some molecular and cellular features of ALS, such as ER stress and MAPK signaling, these pathways are not unique to ALS or PLS and are present in other neurodegenerative diseases, reducing their specificity as biomarkers [52,53].

Furthermore, iPSC-derived neurons do not fully recapitulate all aspects of ALS and PLS pathology, and the complexity of interacting pathways can obscure the identification of direct disease markers. Variations in iPSC generation and differentiation protocols contribute to inconsistent results, complicating cross-study comparisons and hindering the development of universally applicable biomarkers. While iPSC models effectively capture certain cellular vulnerabilities, they often fail to fully elucidate why specific cell types, such as motor neurons, are particularly susceptible in ALS and PLS, limiting their utility in identifying disease-specific biomarkers [52,53].

To summarize, iPSC-derived motor neurons from fibroblasts provide a powerful tool for studying ALS and related neurodegenerative diseases. However, tasks related to variability, incomplete replication of mature neuronal states, and limited specificity emphasize the need for further refinement and validation [52,53].

## 8. Hereditary Spastic Paraplegia (HSP)

The global prevalence of HSP ranges from 0.1 to 9.6 cases per 100,000 people, making it a rare neurodegenerative disorder. HSP is primarily defined by spasticity in the lower limbs and is classified based on inheritance patterns, clinical presentation, and molecular mechanisms. Axonal degeneration commonly appears in the lateral regions of the cervical and thoracic spinal cord, particularly impacting the corticospinal tracts. While advancements in medical diagnostics have improved the ability to identify and manage HSP, current treatments are primarily symptomatic and often do not fully address the needs of patients [54].

### 8.1. Genes Linked to HSP in the Skin

#### *SPAST* and *SPG7* Genes Affect Fibroblasts’ Morphology

A recent study explored the potential of patient-derived skin fibroblasts as biomarkers for examining cell morphology in cases of HSP caused by mutations in the *SPAST* and *SPG7 genes*. Researchers analyzed 100,000 fibroblasts from individuals with these mutations, employing machine-learning techniques to differentiate them from healthy controls. Fibroblasts were selected due to their accessibility and suitability as a non-neuronal model for investigating cellular changes in neurodegenerative diseases. The study revealed that fibroblast morphology varied significantly based on genotype, particularly when exposed to the tubulin-binding drug noscapine. Notably, fibroblasts with SPG7 mutations exhibited a distinct response to the drug, indicating that drug efficacy may depend on the underlying genetic mutation [55].

By combining machine learning and automated image analysis, researchers achieved 100% specificity in distinguishing fibroblasts from healthy controls and those with SPAST or SPG7 mutations. This method also identified genotype-specific drug responses to noscapine with greater sensitivity than traditional approaches, which typically analyze individual cellular features. The findings hold significant clinical implications, as current genetic testing only identifies SPAST mutations in approximately 50% of clinically diagnosed HSP cases. Morphological profiling of fibroblasts could complement genetic testing, serving as a valuable biomarker for diagnosing HSP and predicting drug responses, thereby advancing personalized treatment strategies for neurodegenerative diseases like HSP [55].

However, developing universal biomarkers for HSP using SPAST and SPG7 genes in fibroblasts is challenging due to the distinct morphological and functional differences between SPAST and SPG7 fibroblasts. For example, while noscapine treatment effectively restored acetylated α-tubulin and mitochondrial morphology in SPAST fibroblasts, it had limited efficacy in SPG7 fibroblasts. Although machine learning effectively combines multiple morphological features to differentiate genotypes, overlaps in individual feature values reduce their utility as standalone markers [55].

The analysis of these morphological changes requires advanced imaging and computational techniques, making the process complex and potentially inaccessible for broader clinical applications. Furthermore, the heavy reliance on machine learning for genotype differentiation and drug response analysis complicates reproducibility and standardization across laboratories. Fibroblast models, while valuable, may not fully recapitulate the pathological processes occurring in neuronal cells, which are the primary targets in HSP. This limitation restricts the clinical translation of fibroblast-based biomarkers for diagnosis or disease monitoring [55].

All things considered, fibroblast-based morphological profiling represents a promising approach for diagnosing HSP and predicting drug responses, complementing genetic testing, and contributing to personalized medicine. However, challenges such as scalability, specificity, and the relevance of fibroblast models to neuronal pathology underscore the need for further research and refinement to enhance the clinical applicability of these biomarkers [55].

## 9. Frontotemporal Dementia

FTD is a complicated neurodegenerative condition that causes cognitive decline by affecting the frontal and temporal lobes of the brain, often leading to significant brain atrophy. It is responsible for about 6–8% of early-onset dementia cases. One genetic link to FTD is a mutation in the *CHMP2B* gene, which is associated with FTD dementia type 3 (FTD3). This gene is essential for regulating the endosomal sorting process, which involves trafficking cellular materials to lysosomes for degradation. When mutated, this process is disrupted, leading to cellular dysfunction and contributing to the progression of neurodegeneration. Skin fibroblasts from patients with FTD3 can be reprogrammed into iPSCs, providing an exceptional opportunity to model the disease at a cellular level and potentially identify biomarkers for early diagnosis [12].

### iPSCs

The use of skin fibroblasts from FTD3 (Frontotemporal Dementia with CHMP2B mutations) patients offers a valuable approach for creating patient-specific models of the disease. By reprogramming fibroblasts into iPSCs and differentiating them into cortical neurons, researchers have identified significant disease-related abnormalities. Neurons derived from FTD3 fibroblasts exhibit dysfunctional endosomal activity, impaired mitochondrial structure, reduced mitochondrial function, and increased oxidative stress. Furthermore, these neurons demonstrate disruptions in iron homeostasis, a factor believed to contribute to the neurodegenerative process. These cellular defects, observed in neurons generated directly from patient skin fibroblasts, validate the potential of fibroblasts as biomarkers for identifying the underlying cellular abnormalities in FTD3. Notably, genome editing using CRISPR/Cas9 to correct the CHMP2B mutation reversed many of these abnormalities, underscoring the utility of this approach for studying disease mechanisms and exploring therapeutic interventions [56].

Skin fibroblasts and their iPSC derivatives provide a non-invasive and patient-specific tool for modeling FTD3, enabling researchers to investigate key pathological processes such as mitochondrial dysfunction, oxidative stress, and disrupted iron metabolism. The ability to reverse these disease phenotypes through genome editing further highlights the therapeutic potential of these models. These findings position fibroblast-derived iPSCs as a critical platform for diagnosing and developing targeted treatments for FTD, offering insights into disease mechanisms in a context relevant to the patient’s unique genetic background [56].

iPSC-derived neurons replicate several FTD phenotypes, including endosomal and mitochondrial dysfunction, but they fail to capture the intricate interactions between neurons and glial cells, which are essential for understanding FTD pathogenesis, highlighting significant limitations in their application as biomarkers. Additionally, these neurons often exhibit immature or “fetal-like” characteristics that do not accurately mimic the mature neuronal environment where FTD pathology typically develops. Variability in genotype-specific responses, such as those observed in CHMP2B mutations, complicates the identification of consistent biomarkers across diverse FTD cases. Moreover, many of the observed abnormalities, including mitochondrial dysfunction and oxidative stress, overlap with pathologies of other neurodegenerative diseases, such as Alzheimer’s and Parkinson’s, limiting their specificity as FTD biomarkers [56].

The absence of glial contributions in iPSC models and technical variability in the generation and differentiation protocols further constrain their reliability and reproducibility. These challenges emphasize the need for refinements in iPSC technology and the integration of additional approaches, such as co-culturing neurons with glial cells or combining iPSC data with clinical biomarkers, to enhance the specificity and applicability of these models [56].

Table 4 below outlines the application of fibroblasts as biomarkers in less common neurodegenerative disorders, emphasizing their utility in non-invasive diagnostics, disease modeling, and differentiation of related conditions. 

## 10. Comparison of Fibroblasts and iPS Cells with Brain, Blood, and Retina as Biomarkers for Neurodegenerative Disease

Skin fibroblasts and iPS cells serve as complementary tools for studying neurodegenerative diseases, each offering distinct advantages and limitations. Skin fibroblasts, obtained directly through patient biopsies, retain natural aging-associated features such as mitochondrial dysfunction, oxidative stress, and proteostasis alterations. These cells reflect the donor’s specific aging and genetic background, making them valuable for studying systemic aging processes and biomarkers in diseases like AD and PD. In contrast, iPS cells are derived from somatic cells, such as skin fibroblasts, through a reprogramming process that resets them to an embryonic-like pluripotent state. Although this process erases the aging features inherent in the original cells, iPS cells can be differentiated into various cell types, including neurons and glial cells, enabling detailed modeling of disease-specific mechanisms in tissues most affected by neurodegenerative diseases. However, to replicate aging-related processes in iPS-derived cells, researchers must induce aging-like phenotypes artificially using stressors or genetic modifications [13].

Fibroblasts are advantageous due to their accessibility, ease of collection, and ability to retain systemic aging markers. They provide a natural platform for studying longitudinal changes and systemic biomarkers, such as calcium homeostasis alterations and Erk1/2 phosphorylation in AD. On the other hand, iPS cells require more time and resources but offer unparalleled flexibility for creating disease-relevant models of specific cellular environments. Together, fibroblasts offer insights into natural aging and systemic changes, while iPS cells allow precise modeling of disease pathways in brain-related cells. The integration of both approaches offers a comprehensive framework for advancing neurodegenerative research and developing targeted therapies [13].

When comparing skin-derived models to other tissues used as biomarkers for neurodegenerative diseases, each tissue type has unique advantages and limitations. For example, skin biopsies are non-invasive, retain aging signatures, and enable dynamic studies of mitochondrial function, but they require further validation for disease specificity and may yield conflicting results for biomarkers like α-synuclein in PD. In contrast, brain tissue provides direct assessments of neurodegeneration with high specificity for hallmark pathologies like amyloid plaques, tau tangles, and dopaminergic neuron loss. However, brain tissue biomarkers often require invasive procedures, such as lumbar punctures or autopsy, limiting their applicability for large-scale or longitudinal studies [32].

Blood is highly accessible and suitable for large-scale studies, reflecting systemic inflammation and protein alterations associated with neurodegeneration. However, high systemic variability can reduce specificity, and findings, particularly for amyloid-β, remain inconsistent. Retinal biomarkers offer non-invasive imaging and enable longitudinal monitoring of structural changes that indirectly reflect central nervous system neurodegeneration, such as retinal nerve fiber layer (RNFL) thinning and ganglion cell degeneration. Nonetheless, retinal biomarkers require further validation to ensure consistency across studies and reduce variability in results [32].

In summary, fibroblasts and iPS cells provide distinct yet complementary insights into neurodegenerative diseases, while comparisons with other tissue sources such as the brain, blood, and retina highlight the trade-offs in specificity, accessibility, and practicality. Combining these approaches can yield a more integrated understanding of neurodegeneration and aid in the development of precise diagnostic and therapeutic strategies [13,32].

The following Table 5 provides a comparison of skin with other tissues commonly used as biomarkers for neurodegenerative diseases [32].

## 11. Discussion

**Figure 1:**
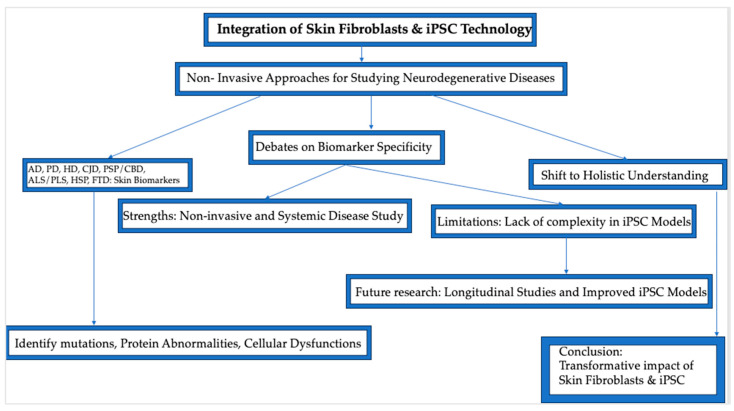
Integration of skin fibroblasts and iPSC technology in neurodegenerative disease research.

The integration of skin fibroblasts and iPSC technology into neurodegenerative disease research has revolutionized our understanding of these disorders. Figure 1 presents a holistic schematic representation of the main points discussed in this review, illustrating this integration. These tools provide non-invasive approaches to identifying genetic mutations, protein abnormalities, and cellular dysfunctions that parallel pathological processes occurring in the brain. Across diseases such as AD, PD, HD, CJD, and tauopathies like PSP, skin biomarkers have proven highly valuable for both research and diagnostics. They provide an initial understanding of disease mechanisms and are instrumental in the development of potential therapeutic interventions. 

There is widespread consensus on the value of skin fibroblasts for studying systemic biomarkers of neurodegenerative diseases. On the one hand, researchers recognize their utility in detecting genetic mutations, such as *APP* and *PSEN1* in AD and *PINK1* and *LRRK2* in PD, as well as identifying protein abnormalities like αSynP and tau protein [3,4,7,8]. In conjunction with this, iPSC technology, which reprograms fibroblasts into neurons relevant to the disease, is seen as a major breakthrough, allowing scientists to model disease progression and test potential treatments [12]. Together, these technologies offer great promise for early diagnosis and provide a non-invasive alternative to more traditional, invasive methods like CSF analysis.

On the other hand, debates and contradictions persist in the literature, particularly regarding the specificity and reliability of certain biomarkers. For instance, while Aβ is commonly found in AD patients, it also appears in healthy individuals, limiting its utility as a definitive marker [16,17,18]. However, its presence in skin tissues suggests that it could serve as an early indicator when combined with other markers and clinical manifestations [19]. Similarly, αSynP, a protein that aggregates in PD, is also observed in other neurodegenerative diseases like LBD and MSA, raising questions about its specificity [37]. Furthermore, while targeting LRRK2 in PD shows promise in reversing mitochondrial dysfunction, the complexity of mitochondrial pathways necessitates a more comprehensive approach tailored to individual patients [34].

The strengths of using skin fibroblasts and iPSCs are significant, primarily due to their non-invasive nature and their ability to reflect systemic disease processes beyond the brain [13]. These tools allow for the study of disease mechanisms without needing to access central nervous system tissues. Still, there are significant limitations. While iPSC models are valuable for replicating many disease characteristics, they frequently fall short of capturing the full complexity of late-onset and sporadic neurodegenerative diseases, reducing their overall applicability [46]. Future research should prioritize larger studies, refined methodologies, and the integration of CNS-derived data to improve the utility of fibroblasts in understanding motor neuron diseases and developing effective diagnostic tools. Conducting longitudinal studies will be crucial for validating the use of skin biomarkers to track disease progression over time [51].

Furthermore, several gaps in the literature persist, particularly about HD. One critical gap is the need for long-term studies that explore the systemic impact of mHTT, which is expressed throughout the body, not just in the brain [5]. While current research has focused primarily on the neurological symptoms of HD, the systemic effects of mHTT on peripheral tissues remain underexplored. For example, the way mHTT impacts organs outside the central nervous system, such as skin fibroblasts, is not yet fully understood [42]. These gaps limit our understanding of the broader pathological effects of HD and hinder the development of comprehensive therapeutic strategies that target both the central and peripheral manifestations of the disease. Significant changes in skin fibroblast biomarkers can be induced via therapeutic interventions, addressing key pathological features of neurodegenerative diseases. Mitochondrial dysfunction and lysosomal impairment, central to PD, may be mitigated through treatments that enhance mitochondrial bioenergetics or lysosomal clearance. For instance, increasing glucocerebrosidase (GCase) activity could reduce lysosomal substrate accumulation, improving cellular homeostasis. Additionally, therapies targeting oxidative stress could lower ROS levels, restoring mitochondrial health. Cellular stress pathways, such as autophagy, protein folding, and trafficking, also exhibit alterations in fibroblasts. Interventions that restore autophagic flux or correct protein mislocalization could normalize these biomarkers, reflecting improved cellular function and offering insights into treatment efficacy [57].

Additionally, combining multiple biomarkers, including genetic markers, protein aggregation, and cellular dysfunctions, could significantly enhance diagnostic accuracy. For instance, integrating αSynP detection with genetic markers such as *PINK1* and *LRRK2* could provide a more holistic and reliable diagnostic framework for PD [4,7]. Moreover, further advancements in iPSC technology are needed to create models that better reflect the complexities of sporadic and late-onset neurodegenerative diseases, which will help in broadening their application across diverse patient populations [46].

Last but not least, the use of skin fibroblasts and iPSCs challenges the traditional view of neurodegenerative diseases as being primarily brain-centric. These technologies demonstrate that many of these diseases affect multiple organs and systems, prompting a more holistic understanding of disease progression [27]. Practically, this shift has profound implications for early diagnosis and the development of personalized medicine, as treatments can be tailored to the unique genetic and cellular profiles of individual patients.

## 12. Conclusions

In conclusion, the integration of skin fibroblasts and iPSC technology represents a transformative shift in the study of neurodegenerative diseases. These tools not only offer non-invasive diagnostic methods but also provide personalized models for exploring disease mechanisms and testing potential therapies. As research continues to evolve, the combination of these technologies with other diagnostic tools and therapeutic strategies will likely lead to earlier diagnosis, more targeted treatments, and, ultimately, improved outcomes for patients suffering from neurodegenerative disorders.

## Figures and Tables

**Table 1:** Comprehensive comparison of biomarkers for Alzheimer’s Disease in skin fibroblasts.

Biomarker	Method of Detection	Sensitivity	Specificity	Key Findings
*APP* (Amyloid Precursor Protein) gene	Gene Expression Analysis, RTQ-PCR	High	High	Mutations in APP are linked to amyloid-β (Aβ) accumulation and are associated with familial early-onset AD [3]
*PSEN1* (Presenilin 1)	Gene Expression Analysis, RTQ-PCR	High	High	Mutations impair amyloid processing, a strong indicator of familial AD, especially early-onset forms [3]
*PPA2* Gene Expression	RTQ-PCR	Medium	Medium	Altered gene expression in AD fibroblasts; reduced phosphatase activity affects tau protein regulation [15]
PKCε Levels	ELISA	High	High	Reduced levels in AD fibroblasts and brain tissue reflecting impaired protective function against Aβ [8]
Amyloid-β (Aβ)	Immunohistochemistry, ELISA	Low	Low	Present in both AD and non-AD patients, limiting its reliability as a standalone AD biomarker [17]
Tau Protein	Seed-Amplification Assay (SAA), RT-QuIC	75–80%	95–100%	Tau seeding activity in skin mirrors brain pathology progression, offering a non-invasive biomarker for AD and other tauopathies [6]
Calcium Homeostasis	Fluorescence Imaging, ELISA	Medium	Medium	Disrupted calcium transport in AD fibroblasts, increasing vulnerability to oxidative stress [32]
Fibroblast Aggregation	Aggregation Rate Calculation	High	High	Higher aggregation rates in AD fibroblasts correlate with disease progression and diagnosis [22]
AD-Index (MAPK Erk1/2)	Phosphorylation Analysis	97%	100%	Measures imbalances in Erk1/2 phosphorylation, accurate for early AD diagnosis using skin fibroblasts [23]
Morphometric Imaging (MI)	3D Matrigel Analysis	High	High	AD fibroblasts form fewer but larger aggregates than controls, aiding in the differentiation of AD from other neurodegenerative conditions [26]
Skin pH	Skin pH Meter	Medium	Medium	AD patients display less acidic skin pH compared to healthy controls, serving as a potential marker [27]
Skin Hydration	Hydration Meter	Medium	Medium	Increased hydration levels in AD patients, distinguishing them from controls [27]
Skin Elasticity	Elasticity Meter	Medium	Medium	Reduced skin elasticity in AD patients compared to healthy individuals, indicating systemic effects [27]
Microvascular Tortuosity	Microvascular Imaging	Medium	Medium	Vascular changes in the skin correlate with cognitive decline, offering early insights into AD [27]
iPSC-Derived Neurons	Reprogramming and Differentiation	High	High	Neurons derived from AD fibroblasts exhibit increased vulnerability to Aβ, providing insights into AD mechanisms [31]

**Table 2:** Phosphorylated α-Synuclein detection in skin biopsies [37]. * N/A: Not applicable.

Condition	% Positive for αSynP	Sensitivity	Specificity
Parkinson’s	92.7%	94%	98%
MSA	98.2%	94%	98%
DLB	96.0%	94%	98%
PAF	100%	94%	98%
Control Group	3.3%	N/A *	N/A

**Table 3:** Comparison of early and recent iPSC models for Huntington’s Disease [46].

Characteristic	Early iPSC Models	Recent iPSC Models
Neuronal Conversion Method	Standard iPSC-derived neurons, free of aggregates and cell death phenotype.	microRNA-based direct conversion to MSNs retains age signatures and exhibits mHTT aggregates, DNA damage, mitochondrial dysfunction, and spontaneous degeneration.
Age-Related Characteristics	Lost during conversion, limiting relevance for age-dependent diseases.	Preserved during conversion, highlighting the role of age in HD progression.
Key HD Features	Often absent (no mHTT aggregates or cell death).	Consistently present: mHTT aggregates, DNA damage, mitochondrial dysfunction, and neuronal degeneration.
Relevance to Late-Onset HD	Limited due to loss of age signatures.	Improved accuracy, as age, is a crucial factor in HD, particularly for late-onset forms.

**Table 4:** Use of fibroblasts as biomarkers in the less common neurodegenerative disorders.

Neurodegenerative Disorder	Fibroblast Biomarker	Role of Fibroblast Biomarkers	Methodology
Sporadic Creutzfeldt–Jakob Disease (sCJD) [10]	Prion-seeding activity detected via RT-QuIC in skin fibroblasts	Alternative diagnostic tool for prion detection in sCJD; potential non-invasive biomarker	RT-QuIC, Western Blot
Progressive Supranuclear Palsy (PSP) [50]	MAPT gene expression (elevated tau isoforms 2N4R, 4R)	Non-invasive method for distinguishing PSP by measuring tau isoforms in fibroblasts	Real-Time PCR, Western Blot
Corticobasal Degeneration (CBD) [50]	MAPT gene expression (elevated tau isoforms 2N4R, 4R)	Fibroblasts reveal tau pathology in CBD, aiding in differential diagnosis	Real-Time PCR, Western Blot
Amyotrophic Lateral Sclerosis (ALS) [51,52]	Gene expression profiling in fibroblasts (RNA processing, hypoxia response)	Fibroblasts show altered gene expression relevant to ALS/PLS, modeling disease mechanisms	Microarray analysis, qPCR, iPSC generation
Primary Lateral Sclerosis (PLS) [51]	Gene expression changes in RNA processing genes	Helps distinguish PLS from ALS through fibroblast gene expression analysis	Microarray analysis, qPCR
Hereditary Spastic Paraplegia (HSP) [55]	Fibroblast morphology profiling (SPAST, SPG7 mutations)	Machine learning identifies genotype-specific fibroblast morphological changes, indicating SPAST and SPG7 mutations	Machine learning, automated image analysis
Frontotemporal Dementia (FTD) [56]	iPSCs from fibroblasts (mitochondrial dysfunction, oxidative stress)	iPSCs derived from fibroblasts display disease-related cellular dysfunction, offering a model for FTD	iPSC generation, CRISPR gene editing

**Table 5:** Comparison of tissues for neurodegenerative disease biomarkers [32].

Tissue	Advantages	Key Biomarkers	Limitations
Skin	Non-invasive, accessible, suitable for long-term studies.	Erk1/2 phosphorylation (AD).	Needs more validation for specific diseases; inconsistent α-synuclein results (PD).
	Reflects aging, oxidative stress, mitochondrial dysfunction.	Altered calcium homeostasis (AD).	
	Allows studies on mitochondrial function and proteostasis.	Impaired mitochondrial function (AD).	
Brain	Highly specific, detects hallmark pathologies (amyloid plaques and tau tangles).	Amyloid plaques and tau tangles (AD).	Invasive (e.g., lumbar puncture), requires autopsy for confirmation, limited for repeated studies.
	Directly assesses central neurodegeneration.	Dopaminergic neuron loss (PD).	
Blood	Easily accessible, non-invasive, ideal for large-scale studies.	PKC levels in RBCs (AD).	High variability; inconsistent findings for some markers like amyloid-β.
	Reflects systemic inflammation and protein alterations.	GSK-3 in WBCs (AD).	
		α-synuclein, DJ-1 isoforms (PD).	
Retina	Non-invasive imaging, allows monitoring of structural changes over time.	RNFL thinning (AD, PD).	Needs more validation for consistency; variability in biomarker results.
	Reflects neurodegeneration indirectly.	Retinal ganglion cell loss (AD).	
	Provides insight into CNS changes.	Retinal dopamine deficits (PD).	

## Data Availability

Not applicable.

## Abbreviations

Central Nervous System (CNS), Alzheimer’s Disease (AD), Parkinson’s Disease (PD), Huntington’s Disease (HD), progressive supranuclear palsy (PSP), Corticobasal Degeneration Disease (CBD), Hereditary Spastic Paraplegia (HSP), sporadic Creutzfeldt–Jakob disease (sCJD), Frontotemporal Dementia (FTD), Amyloid Precursor Protein (APP), Presenilin 1 (PSEN1), Familial AD (FAD), multiple system atrophy (MSA), Peripheral Nervous System (PNS), Amyotrophic Lateral Sclerosis (ALS), Sporadic ALS (SALS), Primary Lateral Sclerosis (PLS), Hereditary Spastic Paraplegia (HSP), Lewy Body Dementia (LBD), A-Synuclein (Asynp), Pure Autonomic Failure (PAF), Pick’s Disease (PiD), Real-Time Quaking-Induced Conversion (RT-QuIC), Real-Time Quantitative Polymerase Chain Reaction (RT-PCR).
